# Efficacy of short-chain polypeptide-based EEN formulas in alleviating intestinal injury in children with Crohn’s disease: a single-center study in China

**DOI:** 10.3389/fnut.2023.931004

**Published:** 2023-05-05

**Authors:** Runqiu Wu, Jin Yang, Jinjin Cao, Peng Wang, Chenhui Wang, Wenxin Chen, Yanling Wu, Xinguo Zheng, Yu Jin, Hui Yang

**Affiliations:** ^1^Department of Pediatric Gastroenterology, Children’s Hospital of Nanjing Medical University, Nanjing, China; ^2^Department of Pediatric Anesthesiology, Children’s Hospital of Nanjing Medical University, Nanjing, China

**Keywords:** short-chain polypeptide, exclusive enteral nutrition, Crohn’s disease, intestinal injury, children

## Abstract

Short-chain polypeptides are composed of three to nine amino acids, which can be absorbed by the intestinal tract without digestive enzymes and ATP energy. Crohn’s disease (CD) is a chronic non-specific disease derived from inflammation and damage of the gastrointestinal tract. In this study, we aim to investigate the effect of short-chain polypeptide-based exclusive enteral nutrition (EEN) formulas on intestinal injury in Chinese children with active CD. From January 2013 to January 2019, a total of 84 consecutive children with a diagnosis of Crohn’s disease (CD) in the Department of Pediatric Gastroenterology, Children’s Hospital of Nanjing Medical University, were divided into mild and moderate-to-severe active CD groups. Each group was further divided into two subgroups: drug group and short-chain polypeptide plus drug group. Tests were carried out on the levels of intestinal fatty acid binding protein (I-FABP) in the blood, fecal calprotectin (FC), and occludin protein in the intestinal mucosa 1 day before treatment and 8 weeks after treatment. Endoscopic and histopathological observations were detected to compare the changes in intestinal injury in children with active CD. After 8 weeks of treatment, the SES-CD scores and Chiu scores of the ileocecal area and terminal ileum of children with mild active CD and the ileocecal area of children with moderate-to-severe active CD in short-chain polypeptide plus drug group were significantly lower than those in the drug group. The OD value of occludin in the terminal ileum and ileocecal area of children with mild active CD and the ileocecal area of children with moderate-to-severe active CD after short-chain polypeptide-based EEN formulas and drug treatment was significantly higher than those in the drug group (*p* < 0.05). Meanwhile, the levels of FC and I-FABP were significantly decreased (*p* < 0.05). The results showed that short-chain polypeptide-based EEN formulas effectively alleviate intestinal injury in children with active CD.

## Introduction

Crohn’s disease (CD) involves chronic relapsing inflammation and damage in the gastrointestinal tract (1). Up to 15% of patients are diagnosed with CD before the age of 20 years (2). Worldwide, the incidence rate of CD in children is between 2.5/100000 and 11.4/100000 (3).

During the occurrence and development of CD, the intestinal epithelial tight junction (TJ) barrier plays an important role (4). A defective intestinal TJ barrier has been implicated as an important pathogenic factor in inflammatory diseases of the gut including CD (4). Occludin protein, as an important part of TJ, is expressed at the top junction between intestinal epithelial cells and regulates the permeability of cell membranes through interaction with the TJ complex (5). The loss or decrease of occludin levels can cause the rupture of TJ, leading to intestinal epithelial TJ barrier dysfunction (6). Intestinal fatty acid binding protein (I-FABP) is a water-soluble protein secreted by monolayer columnar epithelial cells and only exists in gastrointestinal mucosa. After the intestinal mucosa is damaged, I-FABP quickly enters the blood circulation through the cell membrane, capillaries, lymphatic capillaries, and portal vein (7). Therefore, the levels of I-FABP in the blood reflect the degree of intestinal mucosal damage (8).

Severe gastrointestinal injury in pediatric CD causes nutrition and growth retardation, which influences the therapeutic effect and prognosis of children (9). Therefore, early nutrition intervention can promote the improvement of gastrointestinal injury growth and development (10). Although exclusive enteral nutrition (EEN) is one of the effective treatments for pediatric CD and has been recommended as the first-line treatment (11, 12), the influence of different types of nutritional formulas, especially different protein compositions, on the effectiveness of EEN for treatment of active CD is still unclear (13). Short-chain polypeptides are composed of three to nine amino acids, which can be absorbed by the intestinal tract without digestive enzymes and ATP energy. Peptide-based formulas have been shown to improve nitrogen balance and visceral protein synthesis, reduce bacterial translocation and diarrhea, and restore gut integrity (14). Studies have shown that short-chain polypeptides can promote the development of the structure and function of intestinal epithelial cells and contribute to the recovery of the damaged intestinal mucosa (15, 16). However, no clear recommendations are available on the use of short-chain polypeptide-based nutritional formulas in ill children with active CD.

This study aimed to evaluate the effects of drug regimens with or without short-chain polypeptide-based EEN formulas on intestinal injury in pediatric CD patients.

## Materials and methods

### Patients

A total of 114 children with active CD who were hospitalized in the Department of Pediatric Gastroenterology at the Children’s Hospital of Nanjing Medical University, from January 2013 to January 2019, with the diagnostic criteria according to the ESPGHAN revised porto criteria were selected as the research subjects (17). Failure to receive short-chain polypeptide-based EEN feeding according to the protocol resulted in the exclusion of eight patients. In addition, 22 patients were excluded as they did not receive endoscopy after treatment. Finally, 84 consecutive patients, including 41 with mild active CD and 43 with moderate-to-severe active CD were studied. Patients treated with short-chain polypeptide-based EEN formulas (*n* = 42) and without short-chain polypeptide-based EEN formulas (*n* = 42) were considered as the study and control groups. This study was approved by the ethics committee of the Children’s Hospital of Nanjing Medical University, and its clinical trial registration number is Chir 180018278 in Chinese Clinical Trial Registry.

### Inclusion and exclusion criteria

The following inclusion criteria were used to screen children with CD for inclusion in this clinical study: (1) the age of children included was between 1 and 10 years; (2) children who were newly diagnosed as CD; and (3) children with active CD.

The exclusion criteria for children with CD included: (1) the age of children with CD was more than 10 years or less than 1 year; (2) children with CD in remission; (3) children with recurrent CD; (4) children with other chronic intestinal infectious diseases, such as Behcet’s disease, intestinal malignant lymphoma, tuberculosis infection, and hematological diseases; and (5) children with isolated oral ulcers or perianal lesions.

According to the pediatric CD activity index (PCDAI), children with CD were divided into mild active CD (10.0–27.5 points) and moderate-to-severe active CD (≥30.0 points).

### Clinical data

The general information (name, gender, and age), clinical manifestations, and personal and family history were obtained by asking about the patient’s medical history. Clinical and laboratory indicators were collected 1 day before treatment and 8 weeks after treatment and were communicated with the parents. After obtaining the consent of the parents and signing the informed consent form, a colonoscopy (Olympus Lucera cv-260, Japan) was performed under general anesthesia. HE staining and immunohistochemical staining were performed to observe the degree of intestinal mucosal damage and occludin protein expression.

### Short-chain polypeptide-based EEN and drug therapy

Short-chain polypeptide-based EEN formula (Petamen Junior, Nestle Company, Switzerland) was given by oral or nasogastric tube or nasojejunal tube (18). The total amount of short-chain polypeptide-based EEN solution was fed per day according to the children’s weight. The daily amount of nutrient solution for children weighing 1–10 kg, 11–20 kg, and over 20 kg was 100 ml/kg, 1,000 ml + (children weight - 10 kg) × 50 ml/kg, and 1,500 ml + (children weight - 20 kg) × 20 ml/kg, respectively. The energy density was 100 kcal/100 ml. The drug regimens of newly diagnosed mild active CD patients included (19): 2–4 g/day of oral mesalazine for 8 weeks. The drug regimens of newly diagnosed moderate-to-severe active CD patients included (20): corticosteroids were initiated as either 1.5 mg/(kg·day) up to a maximum of 32 mg/day of intravenous methylprednisolone for 1–2 weeks or 1 mg/(kg·day) up to a maximum of 40 mg/day of oral prednisone every morning for 4 weeks, a taper of 40 mg/day of oral prednisone every morning for 2 weeks. An endoscopic reexamination was performed after 8 weeks of treatment.

### Evaluation of intestinal injury under endoscopy

According to the simple endoscopic score for Crohn’s disease (SES-CD), a score of ≤2 was considered as CD endoscopic remission, 3–6 as mild inflammation, 7–15 as moderate inflammation, and ≥ 16 as severe inflammation (21, 22).

### Assessments of intestinal histopathological changes

Colonoscopy was performed before treatment and reexamined after treatment. The cecum and terminal ileum mucosa were stained with HE. Histopathological changes were observed under a light microscope (Olympus Company, Japan). Double-blind scoring was performed by two pathologists according to the modified Chiu’s score standard of small intestinal histopathology (23). The highest score observed in the sample was used to determine the degree of intestinal injury.

### He staining of intestinal tissues

The specimens of ileocecal and ileal tissues were used to prepare the paraffin section. Paraffin section dewaxing to water. The sections were sequentially placed in xylene, anhydrous ethanol, and gradient concentration ethanol for dewaxing. After neutral resin-sealed slides, these samples were observed under a microscope.

### Immunohistochemical staining of intestinal tissues

We took samples of ileocecal colon and terminal ileal mucosa from children with CD for paraffin embedding, sectioning, and immunohistochemical staining for occludin protein. The method was as follows: paraffin sections were dewaxed, hydrated, antigen repaired, and then diluted with occludin working solution (Rabbit anti-human occludin polyclonal antibody, Abcam company, United States). Sheep anti-rabbit IgG (Shanghai Biyuntian Biotechnology Co., Ltd., China) was incubated in a 37°C incubator for 30 min. Streptomyces anti-human occludin peroxidase solution was used to stain, dehydrate, and seal with neutral glue. Finally, 10 randomly chosen visual fields were observed on each section under a microscope for photography. We then used the Image-Pro Plus 6.0 software to determine the average optical density (OD) values of occludin-positive tissues on the staining sections, which is a quantitative occludin protein method and one of the indicators to evaluate intestinal TJ barrier function. In this study, OD values can be used as one of the quantitative values to evaluate the tight junction function of intestinal epithelium before and after CD treatment.

### Determination of I-FABP and FC levels

One day before the endoscopic examination, blood and stool samples were collected from children with active CD, and I-FABP and FC were detected, respectively. After 8 weeks of treatment, we conducted an endoscopic reexamination and collected blood and stool samples again 1 day before the endoscopic examination for I-FABP and FC detection, which were compared and analyzed with those before the examination. The methods of the I-FABP and FC detection were as follows: 2 ml of blood was collected from the elbow vein and placed in a centrifuge for further testing (Beijing Baiyang Medical Equipment Co. Ltd., China). The supernatant was taken, and the I-FABP was determined using the ELISA Kit (Shanghai Xitang Technology Co. Ltd., China). Fecal samples were added to normal saline according to the ratio of 1:50 (mass/volume) and shaken for 20 min. A total of 1 ml of homogenate was placed in the centrifuge, and 0.5 ml of supernatant was taken for detection by the ELISA kit (Wuhan Xinqidi Biotechnology Co. Ltd., China).

### Statistical analysis

SPSS 21 software was used for statistical analysis. The measurement data were expressed by mean ± standard deviation. The comparison between both groups was conducted by *t*-test. The chi-square test was used to compare the count data between groups, *p* < 0.05 indicated that the difference was statistically significant.

## Results

### The effect of short-chain polypeptide-based EEN formulas on intestinal injury of active CD

#### General evaluation of clinical data

There were 41 children with mild active CD, including 21 cases in the drug group and 20 cases in the short-chain polypeptide plus drug group, and 43 children with moderate-to-severe active CD, including 21 cases in the drug group and 22 cases in the short-chain polypeptide plus drug group. There were no significant differences in age, clinical manifestations, and intestinal involvement sites at the same active CD severity between both groups (Table 1).

**Table 1:** Clinical data of children with active CD.

	Mild active CD		*p* value	Moderate to severe active CD		*P* value
	Drug treatment group	Short peptide plus drug treatment group		Drug treatment group	Short peptide plus drug treatment group	
Sample size (*n*)	21	20		21	22	
Male/female	9/12	9/11	0.747	9/12	7/15	0.311
Age (year)	4.62 ± 2.54	3.58 ± 1.00	0.700	5.13 ± 2.60	4.59 ± 2.01	0.400
Positive family history	0 (0%)	1 (5%)	0.311	1 (5%)	1 (5%)	1.000
**Clinical manifestation**						
Abdominal pain	18 (85%)	16 (80%)	0.677	19 (90%)	19 (85%)	0.633
Diarrhea	10 (45%)	10 (50%)	0.757	11 (50%)	13 (55%)	0.752
Hematochezia	5 (25%)	4 (20%)	0.705	5 (20%)	4 (10%)	0.376
Fever	3 (10%)	1 (5%)	0.548	4 (15%)	4 (10%)	0.633
Arthralgia	1 (5%)	0 (0%)	0.311	2(5%)	5 (20%)	0.151
**Montreal classification**						
*Age of diagnosis*						
A1	21 (100%)	20 (100%)		21 (100%)	22 (100%)	
*Site of intestinal involvement*						
L1	6 (29%)	7 (35%)	0.658	5 (24%)	6 (27%)	0.795
L2	3 (14%)	2 (10%)	0.675	2 (10%)	2 (9%)	0.961
L3	14 (67%)	11 (55%)	0.444	14 (66%)	14 (64%)	0.835
*Disease behavior*						
B1	20 (95%)	19 (95%)	0.972	17 (81%)	17 (77%)	0.767
B2	1 (5%)	1 (5%)	0.972	4 (19%)	5 (23%)	0.767
*p*	1 (5%)	1 (5%)	0.972	4 (15%)	4 (15%)	0.942

Short peptide: short-chain polypeptide-based EEN formulas. Drug regimen in mild active CD (oral mesalazine) and moderate-to-severe active CD (intravenous methylprednisolone or oral prednisone).

### The effect of short-chain polypeptide-based EEN formulas on PCDAI score

PCDAI is one of the important indicators to evaluate the severity of disease activity and evaluate the efficacy of pediatric CD. In this study, the PCDAI score of children with mild active CD was 25.50 ± 1.80 in the short-chain polypeptide plus drug group and 25.64 ± 2.03 in the drug group before treatment. There was no significant difference between the two groups (*p* > 0.05). After treatment, the PCDAI score of children in the short-chain polypeptide plus drug group (9.43 ± 2.22) was lower than that in the drug group (11.26 ± 2.64; *p* < 0.05).

For children with moderate-to-severe active CD, the PCDAI score was 56.34 ± 7.06 in the short-chain polypeptide plus drug group and 56.79 ± 9.92 in the drug group before treatment. There was no significant difference between the two groups (*p* > 0.05). After treatment, the PCDAI score of children in the short-chain polypeptide plus drug group (10.91 ± 2.92) was significantly lower than that in the drug group (13.57 ± 2.95) (*p* < 0.05), which suggests that short-chain polypeptide-based EEN feeding reduces disease activity.

### The effect of short-chain polypeptide-based EEN formulas on SES-CD score in intestinal tissues

**Figure 1:**
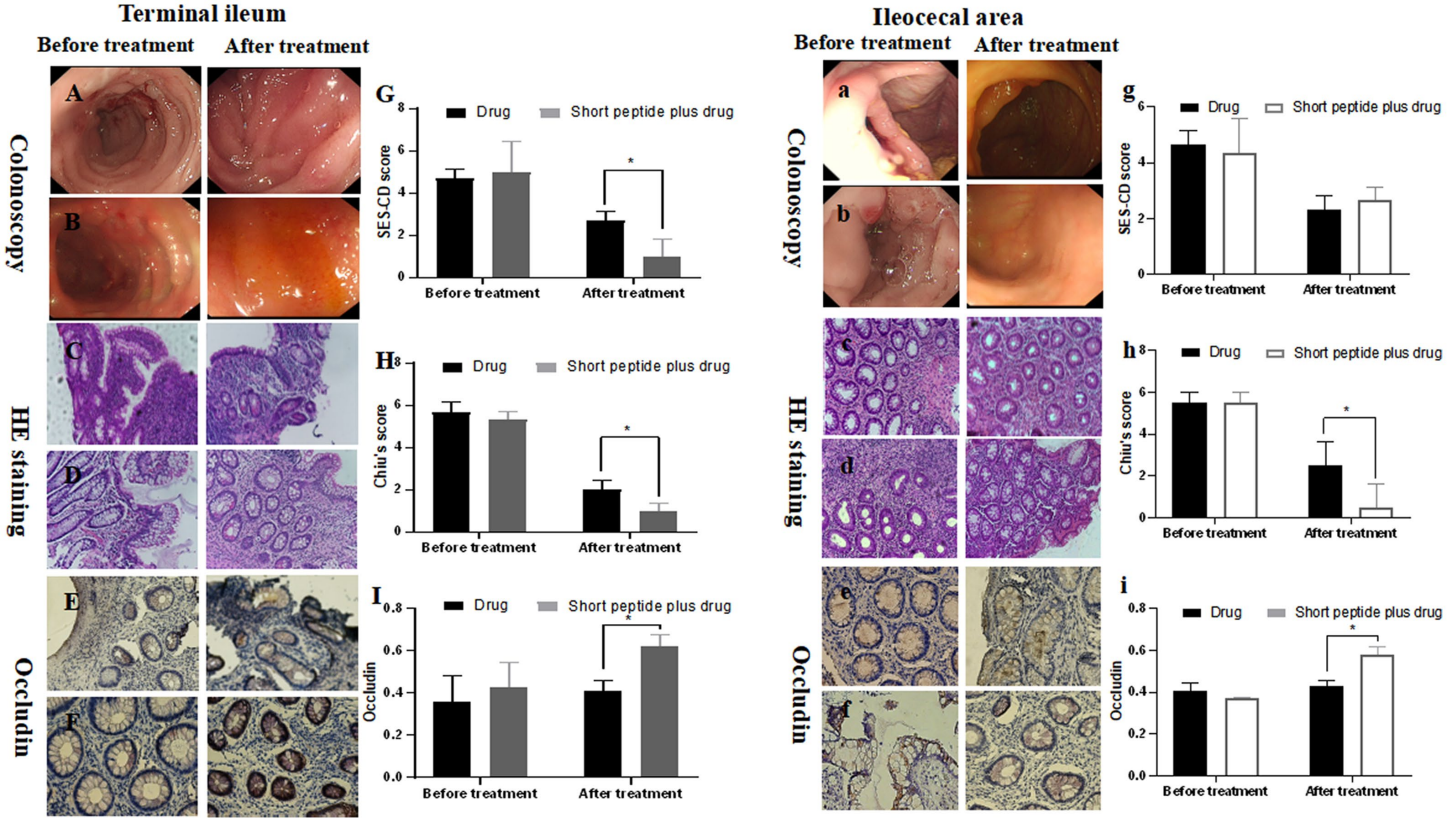
Comparison of SES-CD score, Chiu’s score, and the expression of occludin in ileum mucosa between the drug group and short-chain polypeptide-based EEN plus drug group before and after treatment in children with mild active CD. **(A–I)** Ileum mucosa; **(a–i)** ileocecal mucosa. **(Aa, Cc, Ee)** Drug groups; **(Bb, Dd, Ff)** short peptide plus drug groups. Short peptide: short-chain polypeptide-based EEN formulas. Drug regimen in mild active CD: oral mesalazine. *Compared with the drug group, *p* < 0.05.

Adequate endoscopic scoring in pediatric CD is crucial as an outcome parameter in clinical trials (21). In this study, the SES-CD score of the terminal ileum and ileocecal mucosa was 5.67 ± 0.47 and 4.67 ± 0.47 in the drug group and 5.31 ± 0.41 and 4.33 ± 1.25 in the short-chain polypeptide plus drug group before treatment for children with mild active CD. There was no significant difference at the same site between the two groups. The SES-CD score of terminal ileum mucosa in the drug group after treatment was 2.00 ± 0.41, which was significantly higher than that in the short-chain polypeptide plus drug group (1.00 ± 0.32, *p* < 0.05; Figure 1G). However, there was no significant difference in the SES-CD score of ileocecal mucosa between the drug group (2.33 ± 0.47) and the short-chain polypeptide plus drug group (2.66 ± 0.47) after treatment (Figure 1g).

**Figure 2:**
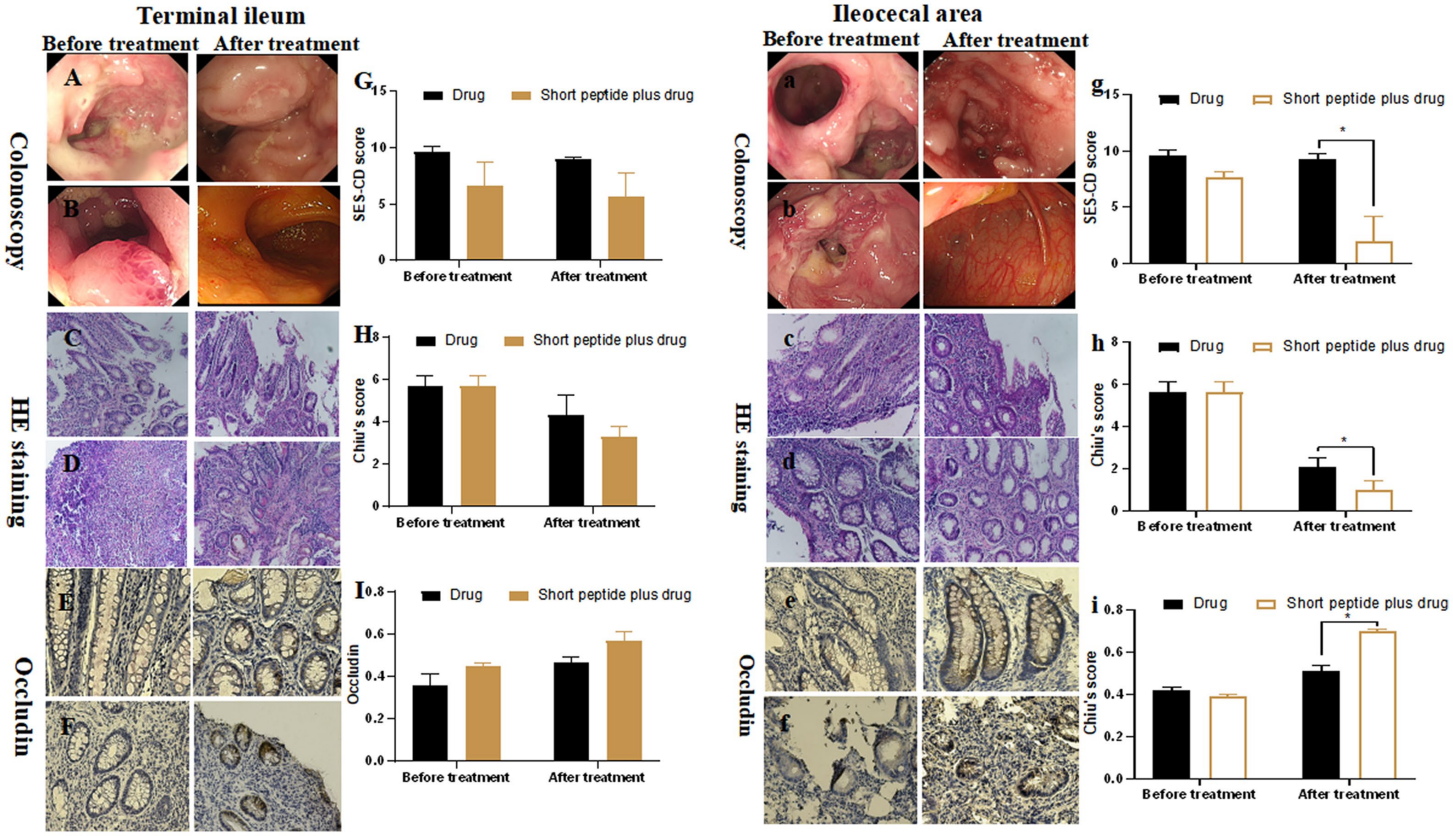
Comparison of SES-CD score, Chiu’s score, and the expression of occludin between the drug group and short-chain polypeptide-based EEN plus drug group before and after treatment in children with moderate-to-severe active CD. **(A–I)** Ileum mucosa; **(a–i)** ileocecal mucosa. **(Aa, Cc, Ee)** Drug groups; **(Bb, Dd, Ff)** short-chain polypeptide-based EEN peptide plus drug groups. Short peptide: short-chain polypeptide-based EEN formulas. Drug regimen in moderate-to-severe active CD: intravenous methylprednisolone or oral prednisone. *Compared with the drug group, *p* < 0.05.

For children with moderate-to-severe active CD, the SES-CD score of terminal ileum mucosa was 9.67 ± 0.47 vs. 9.00 ± 0.15 in the drug group and 6.67 ± 2.05 vs. 2.67 ± 2.05 in the short-chain polypeptide plus drug group before and after treatment, respectively. There was no significant difference between the two groups (Figure 2G). In addition, the SES-CD score of ileocecal mucosa was 9.67 ± 0.47 in the drug group and 7.66 ± 0.47 in the short-chain polypeptide plus drug group before treatment. There was no significant difference between the two groups. After treatment, the SES-CD score of ileocecal mucosa in the drug group (9.33 ± 0.47) was significantly higher than that in the short-chain polypeptide plus drug group (2.0 ± 2.16) (*p* < 0.05; Figure 2g). These results suggest that short-chain polypeptide-based EEN feeding alleviates intestinal mucosal injury of active CD under endoscopic observation.

### The effect of short-chain polypeptide-based EEN formulas on Chiu’s score in intestinal tissues

The results of the pathological analysis of intestinal tissue slices are shown in Figures 1, 2. No significant differences in the degree of histological damage were observed before treatment between the intestinal specimens of the drug and short-chain polypeptide plus drug groups. In addition to the terminal ileum of children in the moderate-to-severe active CD group, intestinal injury in other groups was significantly reduced after treatment compared to before treatment (*p* < 0.05).

For children with mild active CD, the Chiu’s score of the terminal ileum and ileocecal mucosa was 4.67 ± 0.47 and 5.50 ± 0.50 in the drug group and 5.00 ± 1.41 and 5.50 ± 0.50 in the short-chain polypeptide plus drug group before treatment. There was no significant difference at the same site between the two groups. The Chiu’s score of the terminal ileum and ileocecal mucosa in the drug group after treatment was 2.67 ± 0.47 and 2.50 ± 1.11, respectively, which was significantly higher than that in the drug group (1.00 ± 0.82 and 0.50 ± 1.11, respectively, *p* < 0.05; Figures 1H,h).

For children with moderate-to-severe active CD, the Chiu’s score of terminal ileum mucosa was 5.67 ± 0.47 vs. 4.33 ± 0.94 in the drug group and 5.67 ± 0.47 vs. 3.30 ± 0.47 in the short-chain polypeptide plus drug group before and after treatment, respectively. There is no significant difference between the two groups (Figure 2H). The Chiu’s score of ileocecal mucosa was 5.67 ± 0.47 in the drug group and 5.67 ± 0.47 in the short-chain polypeptide plus drug group before treatment. There was no significant difference between the two groups. After treatment, the Chiu’s score of ileocecal mucosa in the drug group (2.10 ± 0.41) was significantly higher than that in the short-chain polypeptide plus drug group (1.00 ± 0.41; *p* < 0.05; Figure 2h). The results suggest that short-chain polypeptide-based EEN feeding alleviates histological damage caused by active CD.

### The effect of short-chain polypeptide-based EEN formulas on the OD values of occludin in intestinal tissues

Furthermore, we tested the expression of the occludin and calculated the OD value in the intestinal tissues (Figures 1, 2). For children with mild active CD, the OD value of occludin in the terminal ileum and ileocecal mucosa was 0.34 ± 0.10 and 0.41 ± 0.04 in the drug group, 0.46 ± 0.02 and 0.37 ± 0.01 in the short-chain polypeptide plus drug group before treatment. There were no significant differences at the same site between the two groups. The OD value of occludin in the terminal ileum and ileocecal mucosa was 0.41 ± 0.05 and 0.43 ± 0.02, respectively, in the drug group after treatment, which was significantly lower than that in the short-chain polypeptide plus drug group (0.62 ± 0.05 and 0.58 ± 0.05, respectively, *p* < 0.05) (Figures 1I,i).

For children with moderate-to-severe active CD, the OD value of occludin in the terminal ileum mucosa was 0.36 ± 0.05 vs. 0.45 ± 0.01 in the drug group and 0.47 ± 0.02 vs. 0.57 ± 0.05 in the short-chain polypeptide plus drug group before and after treatment, respectively. There was no significant difference between the two groups (Figure 2I). The OD value of occludin in the ileocecal mucosa was 0.42 ± 0.01 in the drug group and 0.39 ± 0.01 in the short-chain polypeptide plus drug group before treatment. There was no significant difference between the two groups. After treatment, the OD value of occludin in the ileocecal mucosa (0.51 ± 0.03) in the drug group was significantly lower than that in the short-chain polypeptide plus drug group (0.70 ± 0.01; *p* < 0.05; Figure 2i). In all, the OD value of occludin protein in the intestines after the short-chain polypeptide-based EEN combined with drug therapy was higher than that in the drug group.

### The effect of short-chain polypeptide-based EEN formulas on the levels of I-FABP in the blood and FC

**Figure 3:**
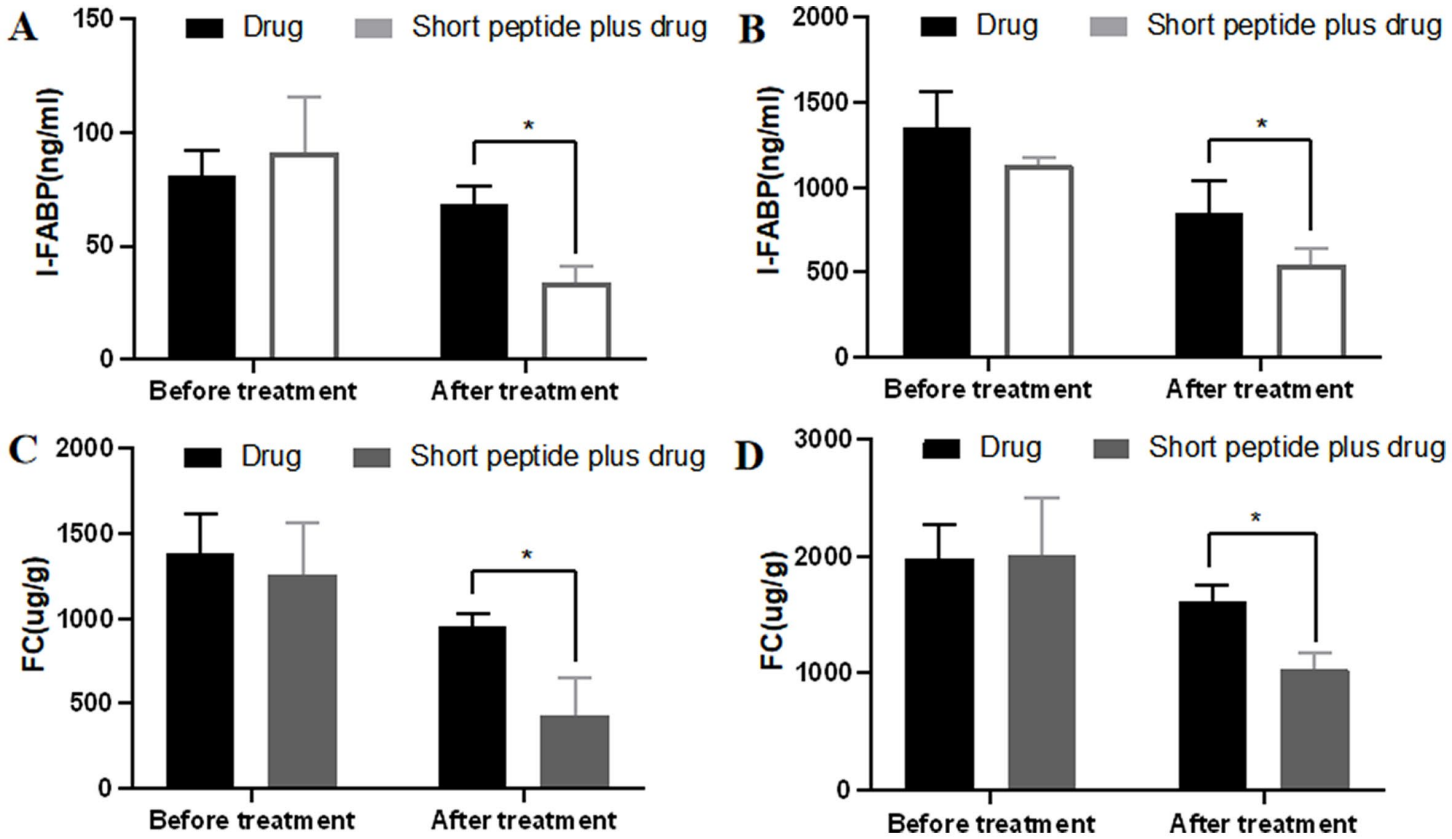
Comparison of the levels of I-FABP and FC between the drug group and short-chain polypeptide-based EEN plus drug group before and after treatment. **(A,C)** Levels of I-FABP in children with mild active CD. **(B,D)** Levels of FC in children with moderate-to-severe active CD. Short peptide: short-chain polypeptide-based EEN formulas. Drug regimen in mild active CD (oral mesalazine) and drug regimen in moderate-to-severe active CD (intravenous methylprednisolone or oral prednisone). *Compared with the drug group, *p* < 0.05.

I-FABP and FC are often used to evaluate inflammatory levels in intestinal injury models. Therefore, we tested I-FABP in the blood and FC in the fecal samples (Figure 3). For children with mild active CD, the concentration of I-FABP and FC was (81.13 ± 11.12) ng/ml and (1389.8 ± 229.97) ug/g in the drug group vs. (91.28 ± 24.15) ng/ml and (1256.82 ± 304.48) ug/g in the short-chain polypeptide plus drug group before treatment. There was no significant difference between the two groups. After treatment, the concentration of I-FABP and FC in the short-chain polypeptide plus drug group [(33.73 ± 7.19) ng/ml and (432.35 ± 218.46) ug/g] was significantly lower than that in the drug group [(68.39 ± 8.07) ng/ml and (956.13 ± 69.68) ug/g, *p* < 0.05; Figures 3A,C]. For children with moderate-to-severe active CD, the concentration of I-FABP and FC was (1349.8 ± 213.95) ng/ml and (1985.26 ± 277.67) ug/g in the drug group vs. (1137.17 ± 38.35) ng/ml and (2017.00 ± 484.23) ug/g in the short-chain polypeptide plus drug group before treatment. There was no significant difference between the two groups. After treatment, the concentration of I-FABP and FC in the short-chain polypeptide plus drug group [(542.21 ± 97.51) ng/ml and (1038.28 ± 130.3) ug/g] was significantly lower than that in the drug group [(854.69 ± 189.72) ng/ml and (1616.41 ± 133.54) ug/g, *p* < 0.05; Figures 3B,D]. The findings show that lower levels of inflammation occurred in the short-chain polypeptide plus drug group.

## Discussion

The main therapeutic goal of pediatric CD is to induce and maintain clinical remission and mucosal healing, promote growth and development, and improve quality of life in children (24). It is increasingly important to accurately quantify the measurable concepts, including children-reported symptoms, intestinal damage and transmural inflammation, histologic appearance, as well as quality of life, disability, and other patient-centered attributes (25). Standardized indices, which show sufficient validity, reliability, and responsiveness to change are not only mandatory for implementing the treat-to-target approach but are also critical for assessing the effectiveness of emerging medications (25). Certain indicators can be accurately assessed through the use of existing measurement methods including PCDAI, SES-CD, Chiu’s score, and FC and I-FABP.

PCDAI, as one of the pediatric indices and scales for assessing disease activity, is well established. In this study, the score of PCDAI in both the mild and moderate-to-severe CD groups decreased significantly after short-chain polypeptide-based EEN was combined with drugs. It suggests that the short-chain polypeptide-based EEN combined with drug regimens has more efficacy in reducing the disease activity of pediatric active CD than that of drug regimens alone.

Healing of mucosal lesions is an important endpoint in clinical trials of treatments for pediatric CD. Mucosal healing has been proposed as a new target of pediatric CD therapy. Nowadays, the CD endoscopic index of severity is the only validated endoscopic activity score. However, this index has the disadvantage of being complex and time-consuming, which limits its application in clinical practice (26). Thus, the simple endoscopic score for SES-CD as a widely used index is suitable for evaluating clinical and endoscopic activity (21). Furthermore, intestinal histopathological changes detected by HE staining and Chiu’s scores are used to make more accurate assessments of the severity of the intestinal injury, which is currently the most commonly used method (23). This study found that, regardless of whether terminal ileum or ileocecal area, after treatment of short-chain polypeptide-based EEN combined with drugs, the endoscopic score for SES-CD and histopathological Chiu’s scores decreased significantly in children with mild CD. While the same changes occurred in the ileocecal region of the lesion in children with moderate-to-severe active CD. It was shown that short-chain polypeptide-based EEN feeding can promote mucosal healing and induce and maintain CD remission, which is obvious in children with mild CD. For children with moderate-to-severe CD, the degree of recovery of the terminal ileum and ileocecal area is significantly different. It is speculated that the recovery speed of intestinal barrier function varies with the location where CD involves the intestines. Additionally, we found that the improvement of SES-CD endoscopic score and histopathological Chiu score in the ileocecal area of children with moderate-to-severe active CD was significantly better than that of children with mild active CD, which suggests that the degree of recovery of intestinal mucosal injury in the ileocecal area of children with moderate-to-severe CD was significantly higher than that of children with mild CD. It is speculated that the possible reason for this profound difference is that methylprednisolone or prednisone is the combined drug for the treatment of children with moderate-to-severe active CD, which is more effective than mesalazine for mild active CD treatment in inducing remission and promoting mucosal healing.

The intestinal epithelial cell apoptosis and the dysfunction of the intestinal epithelial barrier are the principal reasons for the increased intestinal permeability in CD (27). The main function of the intestinal epithelial barrier is to maintain intestinal permeability, which is formed by the dynamic changes of TJ and responds to different extracellular stimuli. Occludin can regulate the formation of TJ through protein–protein interaction (28). Previous studies have shown that injury to intestinal epithelial TJ is the leading cause of CD onset with underexpression of TJ-related proteins including occludin (29). In this study, the OD values of occludin in the terminal ileum and ileocecal area of the mild active CD and ileocecal area of moderate-to-severe active CD after short-chain polypeptide-based EEN feeding combined with drug treatment were significantly higher than those in the drug group, which suggests that the short-chain polypeptide-based EEN combined with drug regimens have more efficacy in alleviating gut damage and promoting mucosal healing of active CD than that of drug regimens alone.

FC is released by innate immune cells activated during cellular stress and injury (30). A prospective cohort study showed that the sensitivity and specificity of FC in CD were 100 and 97%, respectively (31). The study also found that the FC level in colonic active CD was significantly higher than that in ileal active CD (32). Sipponen et al. (33) showed that infliximab after 12 weeks of treatment had a significant decrease in FC, which is related to the endoscopic severity index (γ = 0.561, *p* = 0.03), indicating that FC is related to mucosal healing under endoscopy. In this study, there were no significant differences in the FC level between the two groups before treatment. However, the FC level was sharply lower in the short-chain polypeptide-based EEN plus drug group than that in the drug group, suggesting that short-chain polypeptide-based EEN feeding can promote the intestinal mucosal healing of children with active CD.

I-FABP is a type of low molecular weight (15 kDa) intracellular protein that plays a role in fatty acid transport and metabolism (34). I-FABP presents a gradient distribution in the mature small intestinal mucosa, the content of villi is higher than that of lacuna and the content of the proximal and middle 1/3 of the jejunum is higher than that of the distal 1/3 of the jejunum (35). Murat et al. showed that I-FABP is a useful systemic marker for CD activity (36). In this study, there were no differences between the two groups before treatment despite whether the children had mild or moderate-to-severe active CD. However, I-FABP levels in the blood significantly decreased after short-chain polypeptide-based EEN formulas combined with drug treatment compared with that in the drug group. The reason may be that short-chain polypeptide-based EEN feeding promotes the recovery of the surface of the intestinal mucosal villi, which is easily affected by active CD and reduces the release of I-FABP into the peripheral blood.

## Conclusion

The study found that short-chain polypeptide-based EEN formulas effectively alleviate gut damage in children with active CD. When they are combined with drug regimens, they show more efficacy than drug regimens alone. Short-chain polypeptides can be absorbed by the intestinal tract without digestive enzymes and ATP energy, contributing, in particular, to the recovery of damaged intestinal mucosa of pediatric CD. Therefore, short-chain polypeptide-based EEN formulas should be recommended for the induction of remission in children with newly diagnosed active CD. Due to the limitations of a single center, a small sample size, and the lack of long-term follow-up data, it is necessary to conduct a multicenter prospective randomized trial with a larger sample size and extend the follow-up observation time to provide definite evidence to establish the extent of the benefits.

## Data availability statement

The original contributions presented in the study are included in the article/supplementary material, further inquiries can be directed to the corresponding authors.

## Ethics statement

This study was approved by the ethics committee of children’s Hospital of Nanjing Medical University. Written informed consent to participate in this study was provided by the participants’ legal guardian/next of kin. Written informed consent was obtained from the minor(s)’ legal guardian/next of kin for the publication of any potentially identifiable images or data included in this article.

## Author contributions

HY and YJ conceived of the idea and were responsible for the planning, content, and structure of the article. HY wrote the initial manuscript draft. RW, JY and JC performed clinical case observation, data collection and analysis. PW, CW, WC, YW and XZ participated in the collection of intestinal tissue samples and immunohistochemical experiments, data collection and analysis. All authors contributed to the article and approved the submitted version.

## Funding

The study was supported by grants from the Scientific Development Project of Nanjing City (no. 201605045), the Medical Science and Technology Development Project of Nanjing City (YKK21153), and the Science and Technology Development Project of Nanjing Medical University (NMUB2020101).

## Conflict of interest

The authors declare that the research was conducted in the absence of any commercial or financial relationships that could be construed as a potential conflict of interest.

## Publisher’s note

All claims expressed in this article are solely those of the authors and do not necessarily represent those of their affiliated organizations, or those of the publisher, the editors and the reviewers. Any product that may be evaluated in this article, or claim that may be made by its manufacturer, is not guaranteed or endorsed by the publisher.
